# Reply to Ghobadi and Jafari: diet quality in relation to the risk of hypertension among Iranian adults: cross-sectional analysis of Fasa PERSIAN cohort study

**DOI:** 10.1186/s12937-024-00988-4

**Published:** 2024-07-24

**Authors:** Amir Motamedi, Maryam Ekramzadeh, Ehsan Bahramali, Mojtaba Farjam, Reza Homayounfar

**Affiliations:** 1 Student Research Committee, Shiraz University of Medical Sciences, Shiraz, Iran; 2 Nutrition Research Center, Department of Clinical Nutrition, School of Nutrition and Food Sciences, Shiraz University of Medical Sciences, Shiraz, Iran; 3 Noncommunicable diseases research center, Fasa university of medical sciences, Fasa, Iran; 4 Faculty of Nutrition and Food Technology, National Nutrition and Food Technology Research Institute, Shahid Beheshti University of Medical Sciences, Tehran, Iran

## Abstract

Ghobadi and Jafari have mentioned some points about our article titled “Diet quality in relation to the risk of hypertension among Iranian adults: cross-sectional analysis of Fasa PERSIAN cohort study” which was published in the Nutrition Journal. Thanks for their consideration, the following is provided as a response to their comments.

## Main text

To the editor:

We have meticulously studied the letter from Ghobadi and Jafari [1] about our recent article on diet quality in relation to the risk of hypertension among Iranian adults published in the Nutrition Journal [2]. We believe that there were misinterpretations and misunderstandings of the research design and results.

Regarding their concern about the Food Frequency Questionnaire (FFQ), the 125-item FFQ used in this study is a short-form (Just reduce the number of food items) of the questionnaire originally validated for the Golestan cohort study in Iranian population [3], which is a Willett-type questionnaire in format [4] with essential modifications for type of food and number of food items based on the Iranian dietary patterns. Therefore, we did not use the questionnaire validated by Willett et al. [4] to assess the dietary intake of participants. Previous studies published from the Fasa PERSIAN cohort study [5, 6] have used the 125-item FFQ reported in our study. The validity study of the existing questionnaire has also been done and its article is under review for publication (unpublished data).

Several current guidelines suggest to adjust the analyzes for a few pre-specified confounders to evade possible bias in estimation of the associations because of post-hoc choice of confounders and “fishing” for confounders that influence the significancy of the statistical analyzes [7]. Confounders should commonly be selected according to their expected capability to predict outcomes, irrespective to whether they present “imbalances” (i.e., irrespective to whether there is remarkable difference in distribution of a specific variable between the group with hypertension and the group without hypertension). On the other side, adjustment for confounders would be most useful when the confounders are toughly prognostic for the outcome [8]; accordingly, adjustment for independent variables with a P-value less than 0.2 is not an optimal strategy to control the results for confounders; this approach results in an over-adjustment bias [9]. In our study, pre-specified confounders were selected for adjustment in binary logistic regression analysis based on the previously published studies in this area of research [10, 11]. Noteworthy, further adjustment for drugs influencing blood pressure did not remarkably change the results. The authors also mentioned that it is expected that crude effect size in multivariable models changes by at least 10% after adjusting covariates [1], but there is no crude model in our study; the model 1 is adjusted for daily energy intake.

The mean daily energy intake in women with and without hypertension was 2842.17 ± 1058.11 and 3007.81 ± 1048.50 kcal/d respectively (*p* = 0.001). For men, the mean intake of energy was 3821.23 ± 1435.75 in subjects with hypertension and 3922.29 ± 1058.11 in subjects without hypertension (*p* = 0.05). These findings are comparable with the results from other published studies on the Fasa PERSIAN cohort study [12]. It should be considered that dietary intake is remarkably various in different sub-populations. Hence, we agree that the mean daily energy intake of the participants of Fasa PERSIAN cohort study may be higher than other populations which may be justified considering the fact that the demographic composition of the area is rural and the occupation of the majority of people is agriculture.

## Data Availability

Not applicable.
